# Large-Scale Synthesis of Carbon Nanomaterials by Catalytic Chemical Vapor Deposition: A Review of the Effects of Synthesis Parameters and Magnetic Properties

**DOI:** 10.3390/ma3084142

**Published:** 2010-07-30

**Authors:** Xiaosi Qi, Chuan Qin, Wei Zhong, Chaktong Au, Xiaojuan Ye, Youwei Du

**Affiliations:** 1 Nanjing National Laboratory of Microstructures and Jiangsu Provincial Laboratory for NanoTechnology, Nanjing University, Nanjing 210093, China; E-Mails: xiaosi6460099@yahoo.com.cn (X.S.Q.); icecat1988@gmail.com (C.Q.); lcsyxj@163.com (X.J.Y.); dyw@nju.edu.cn (Y.W.D.); 2 Chemistry Department, Hong Kong Baptist University, Hong Kong, China

**Keywords:** carbon nanomaterials, catalytic chemical vapor deposition, magnetic properties, microwave absorbing ability

## Abstract

The large-scale production of carbon nanomaterials by catalytic chemical vapor deposition is reviewed in context with their microwave absorbing ability. Factors that influence the growth as well as the magnetic properties of the carbon nanomaterials are discussed.

## 1. Introduction

**Figure 1:**
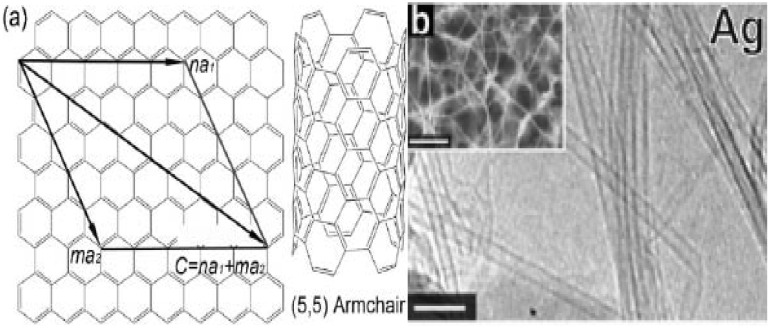
**(a)** Formation of single-walled nanotubes (SWNTs) by rolling a graphene sheet along a chiral vector c [17] and **(b)** Transmission electron microscopy (TEM) and Scanning electron microscopy (SEM) (inset) micrographs of SWNTs grown on Ag [15].

Since the landmark paper of Ijima [1], carbon nanotubes (CNTs) have been studied widely [2,3,4]. The unique physical and chemical properties of CNTs suggests that the materials can potentially be utilized in areas such as field emission display [5], microelectronic devices [6,7,8,9,10,11,12], hydrogen storage [13] and composite material additives [14]. Generally, CNTs can be divided into two categories: single-walled nanotubes (SWNTs) [15] and multi-walled nanotubes (MWNTs) [16]. The molecular structure of SWNTs can be visualized as graphene sheets that roll up in a particular direction as designated by a pair of integers (Figure 1) [16,17]. Because of the quasi one-dimensional (1D) structure, SWNTs display unusual mechanical, electrical, optical, chemical and thermal properties [18,19,20,21,22] that are ideal for nano-electronic devices such as quantum wires [23], single electron transistors [24] field-effect transistors and sensors [25,26]. In the past two decades, SWNTs were synthesized by laser ablation [27] or arc discharge method [28]. For the fabrication of SWNT-based devices, the as-synthesized SWNTs have to be purified and then suspended in an organic solvent for final deposition onto a selected substrate. One can envisage that precision control on the orientation of SWNTs for device integration is difficult. When one comes to commercialization, one has to produce pure and well-defined SWNTs in low cost. If one wants to adopt the existing processes in semiconductor industries for SWNT production, a route with temperature much lower than that of laser ablation and arc discharge is required [29]. One of the approaches that can satisfy such a requirement is chemical vapor deposition (CVD).

**Figure 2:**
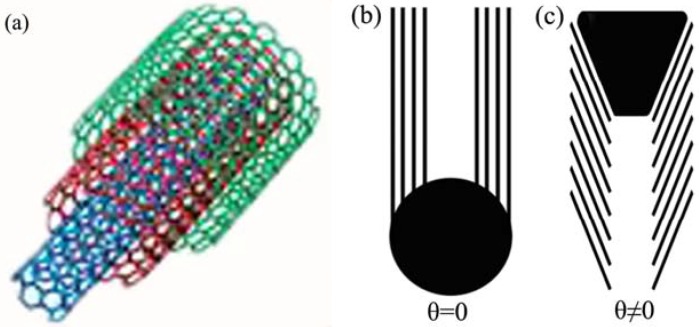
**(a)** Formation of multi-walled nanotubes (MWNT) by rolling up a stack of graphene sheets into concentric cylinders; straight MWNTs **(b)** θ=0 and **(c)** θ≠0 [30].

Similarly, MWNTs can be considered as a stack of graphene sheets that roll up into concentric cylinders (Figure 2a), with each wall layer (a graphite basal plane) parallel to the central axis (θ=0) (Figure 2b). When the angle between the graphite basal planes and the tube axis is nonzero, stacked-cone structures similar to that of bamboo or piled cone result (Figure 2c) [30].

**Figure 3:**
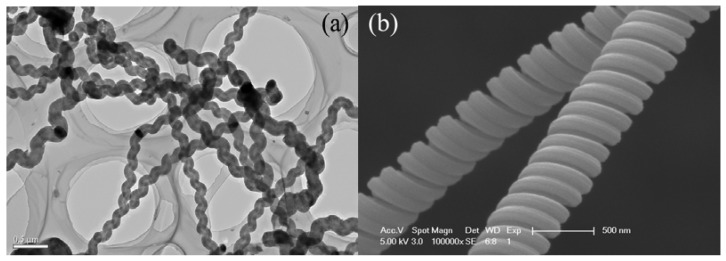
**(a)** TEM image of helical carbon nanotubes (HCNTs) and **(b)** FE-SEM image of double carbon nanocoils (CNCs) [34].

Moreover, due to the existence of hexagonal, pentagonal and heptagonal carbon rings, CNTs with shapes unlike that of straight CNTs, such as helical carbon nanotubes (HCNTs) [31] and carbon nanocoils (CNCs) [32], are formed. A HCNT is constructed by periodically introducing heptagonal and pentagonal rings into the hexagonal network of the graphene layers along the tube axis [33]. Unlike the linear carbon nanofibers (CNFs) and CNTs, HCNTs (Figure 3a) and CNCs (Figure 3b) [34] are chiral materials. If an electrical current passes through a HCNT or CNC, there is the generation of an inductive magnetic field. Therefore, HCNTs and CNCs are potentially applicable in the fabrication of electromagnetic nanoswitches and nanotransformers. In addition, micro/nano carbon coils have the potential of being used as nanosprings [35], micro-antenna, bio-activators or bio-deactivators, energy converters [36], micro-sensors [37], *etc*.

Depending on where the pentagonal and heptagonal rings are placed, HCNTs can be metallic, semiconducting or semi-metallic, which is impossible for the straight CNTs [38]. In arc-discharge and laser ablation processes, straight CNTs are formed owing to the high temperature (required for graphite vaporization) and the high mobility of carbon atoms. However, in CVD processes, CNTs with straight or curved morphologies are often formed simultaneously. For example, HCNTs or CNCs were reported as by-product by Ivanov *et al.* in the synthesis of CNTs by means of CVD [39]. In the past 20 years, efforts have been put in to synthesize HCNTs and CNCs selectively [39,40,41,42,43,44,45,46,47,48,49,50]. The texture of catalytically grown CNCs [41] and the growth mechanism of HCNTs [42] were studied. By optimizing experimental parameters [45,49] and using specific catalysts [46,47,48,49,50], one can enhance the selectivity to HCNTs or CNCs. Hou *et al*. [46] reported the synthesis of HCNTs in the co-pyrolysis of Fe(CO)_5_ (as floating catalyst precursor) and pyridine or toluene (as carbon source) in H_2_ at temperatures above 1000 °C. Luo *et al.* [51] synthesized HCNTs as major products in the thermal reduction of ethyl ether over Zn in a stainless-steel autoclave at 700 °C. Bajpai *et al*. [44] synthesized perpendicularly aligned HCNTs by the co-pyrolysis of Fe(CO)_5_ and pyridine in a mixed flow of Ar and H_2_ at temperatures ranging from 900 to 1100 °C. Motojima and co-workers [52,53] synthesized double-helix CNCs over nickel through a high-temperature route, and reported that the Ni particles were often located at the tips of the CNCs. Pan *et al*. [54] showed that the uneven speed of carbon extrusion at different parts of the catalyst grains led to the helical growth of coils.

**Figure 4:**
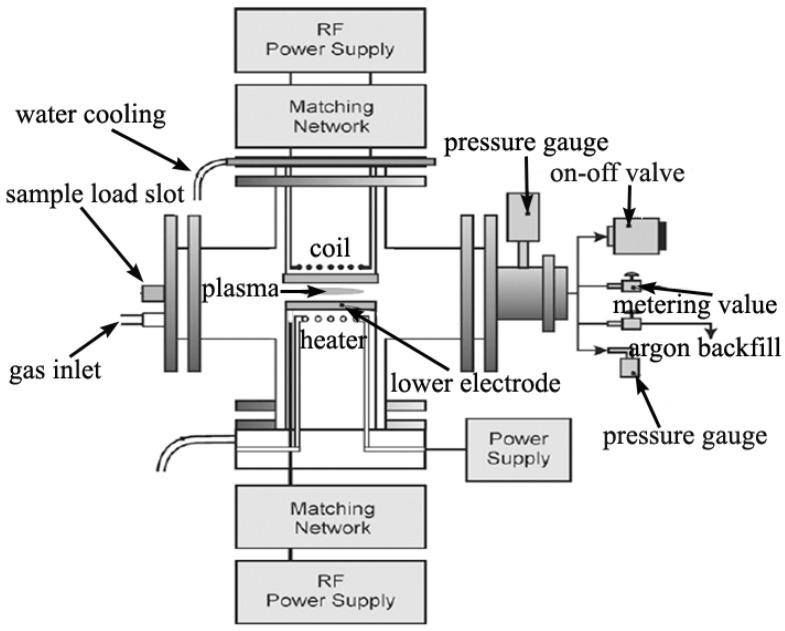
Schematic of a typical plasma-enhanced chemical vapor deposition (PECVD) setup [30].

In terms of upward scalability, cost, and synthesis temperature, the CVD method is superior to arc discharge and laser ablation. The plasma-enhanced CVD (PECVD) method can be used to synthesize carbon nanomaterials in large scale. An advantage of using the PECVD approach is that the as-synthesized products are almost totally deposited on the substrate and are easy to collect. At present, this is the only technique that allows size, alignment and orientation control of nanospecies [55]. The plasmas used to decompose and activate the reactants in the gas phase are usually generated by hot filaments (HF) or by electrical discharges at different frequencies (DC, RF, and MW). The schematic of a typical PECVD set-up is shown in Figure 4 [30]. In PECVD synthesis, the catalyst powder was generally loaded on a substrate by means of wet chemistry or sputtering, usually accompanied by either chemical etching or thermal annealing to induce the formation of catalyst particles on the substrate. During synthesis, the substrate was kept at temperatures in the range of 650–1500 °C, and reactant gas was introduced into the reactor with a pressure typically below 100 Torr. Moreover, the catalysis could be homogeneous in PECVD processes. For example, a metallorganic compound (e.g., ferrocene) was introduced into the reactor and decomposed by the plasma, directly providing metallic clusters in the gas phase [30].

Nanomaterials can also be synthesized by means of thermal CVD. The method makes use of a flow reactor (quartz tube or stainless steel tube) placed inside a furnace for the pyrolysis of carbon-containing molecules. As reactants, carbon-rich gases mixed with argon or nitrogen flows are used, but sometimes the reactant can be in the form of liquid that is subsequently vaporized. If a catalyst is involved, the method is called catalytic CVD (CCVD). The catalyst can be either in powder form driven into the reactor together with the feed gases or previously coated or supported on a substrate. It is known that interesting and novel processes can occur under high-pressure conditions. Nevertheless, atmospheric pressure is often adopted for easy management. Compared to the PECVD method, the CCVD method has higher scalability but poorer control in deposition area. It is known that large-scale generation of a material is a critical factor for commercialization. Thus, wide utilization of carbon nanomaterials depends on whether they can be produced in large scale efficiently. As indicated by many studies [44,46,51], the CCVD approach is suitable for large-scale production of carbon nanomaterials.

**Figure 5:**
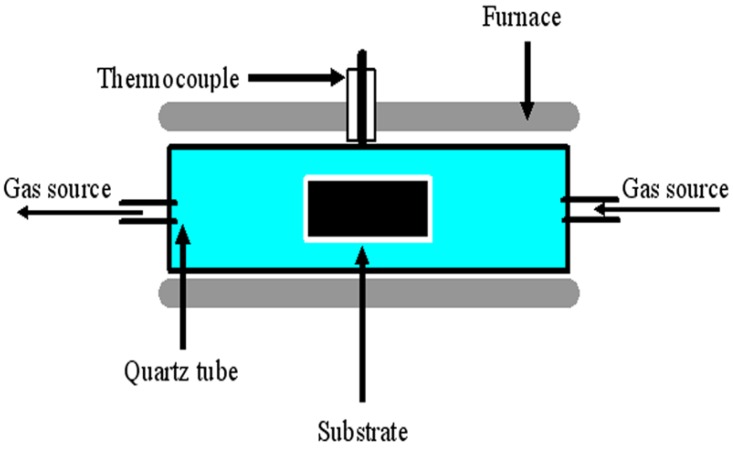
Schematic of a typical thermal CVD apparatus.

In Figure 5, the schematic of a typical thermal CVD apparatus is shown. By means of catalytic decomposition of hydrocarbons, magnetic materials can be encapsulated inside carbon nanomaterials. There are potential applications of carbon nanospecies that are decorated with magnetic materials. For example, magnetic CNTs in the form of capsules or nanosubmarines can be used in the delivery of drugs to a desired location of a human body [56] and magnetic HCNTs can be used as a good microwave-absorbing material [57]. Generally speaking, the morphology and quality of the as-synthesized carbon species depend on factors such as (i) selection of catalyst and carbon source, (ii) temperature for the reduction of catalyst precursor, (iii) temperature for the pyrolysis of carbon source, (iv) nature of catalyst support, and (v) impurity in catalyst. Currently, the carbon sources for CCVD studies are acetylene, methane, carbon monoxide, ethylene, alcohols, toluene, pyridine, *etc.* After much research works, scientists find that carbon nanomaterials such as carbon nanospheres (CNPs), carbon nanobelts (CNBs), carbon nanorods (CNRs) can also be synthesized in CCVD processes by regulating the reaction parameters, giving good control in the morphology, scale, and selectivity of a desired product. In this article, we review the parameters that affect the quality of carbon nanospecies synthesized in CCVD processes, making emphasis on the mass production of carbon nanomaterials of different structures.

## 2. Results and Discussion

### 2.1. Effect of Reactor Setup

**Figure 6:**
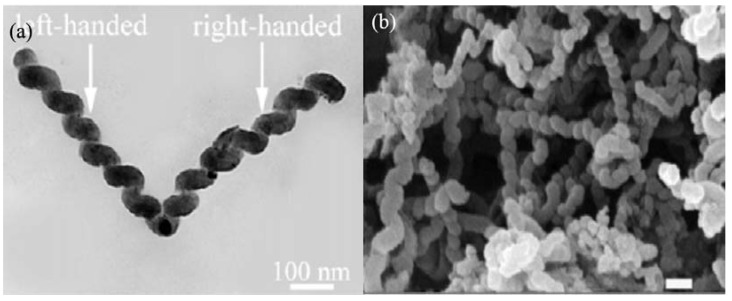
Typical **(a)** TEM and **(b)** FE-SEM image of helical carbon nanofibers (HCNFs) synthesized in a stainless steel reactor over Fe nanoparticles generated by a combined sol-gel/reduction method [60].

**Figure 7:**
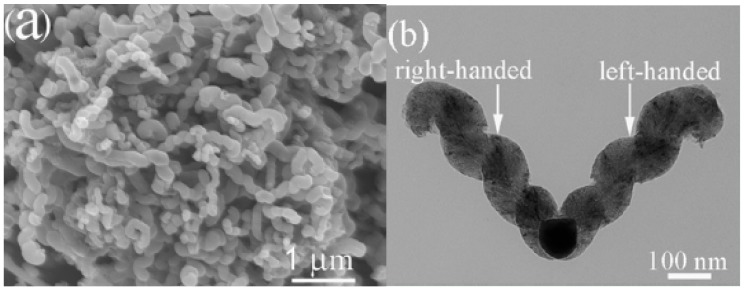
Typical **(a)** FE-SEM and **(b)** TEM images of helical carbon nanofibers (HCNTs) synthsized in a quartz reactor over Fe nanoparticles generated by a combined sol-gel/reduction method [57].

As shown in Figure 5, a typical thermal CVD setup for the synthesis of carbon nanomaterials consists of a reactor, gas source, thermocouple, tube furnace and substrate (where the catalyst is located). Commonly, a reactor is made of quartz or stainless steel in the form of a tube. It was noted that CVD reactivity inside a stainless steel reactor could be significantly different from that inside a quartz one. For example, a higher yield was observed in the former [58], whereas better selectivity was observed in the latter [59]. These differences are understandable, because iron is the major element in stainless steels, and elements such as Fe, Co, Ni and their alloys are known catalysts in CCVD processes. It is apparent that the iron in stainless steels has a positive effect on the product yield. Similar phenomena were observed by us [57,60]. Using a stainless steel tube, we found that the product of acetylene pyrolysis over Fe nanoparticles at 450 °C was helical carbon nanofibers (HCNFs) (1.582 g, mass of product collected after reaction) [60]. As shown in Figure 6, the HCNFs have two coiled nanofibers grown on a catalyst nanoparticle. The two coils are identical in cycle number and are almost equal in diameter, length, and pitch; the coiling direction, however, is opposite. In a quartz tube with the other factors kept unchanged, we observed that the product is HCNTs (85% selectivity) and in much lower quantity (0.413 g) (Figure 7a). As shown in Figure 7b, the HCNTs have two coiled nanotubes grown on a catalyst nanoparticle. The two tubes are mirror images to each other and are coiled in a regular and tight fashion with very short coil pitches [57].

**Figure 8:**
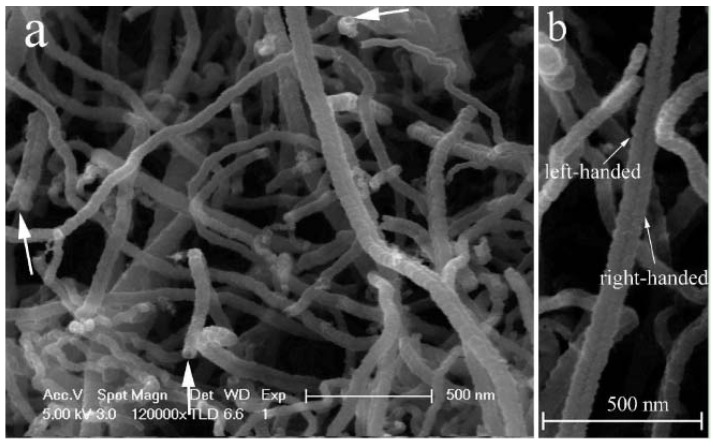
FE-SEM images of plait-like carbon nanocoils (CNCs) collected inside a quartz reactor: **(a)** Low magnification with white arrows indicating openings of tubes; **(b)** plait-like CNC with two CNCs fused together in opposite handedness; the left one is left-handed while the right one right-handed [61].

When Ni nanoparticles were used as catalyst, we found that the product collected in acetylene pyrolysis at 415 °C inside a quartz tube was different from that collected inside a stainless steel tube [61]. The product collected inside a quartz reactor is crystalline plait-like CNCs (3.032 g) (Figure 8a). As shown in Figure 8b, one can see that the plait-like structure exhibits two CNCs of opposite handedness growing alongside each other in a fused manner; the two CNCs are almost identical in diameter, length, pitch, and coil number.

**Figure 9:**
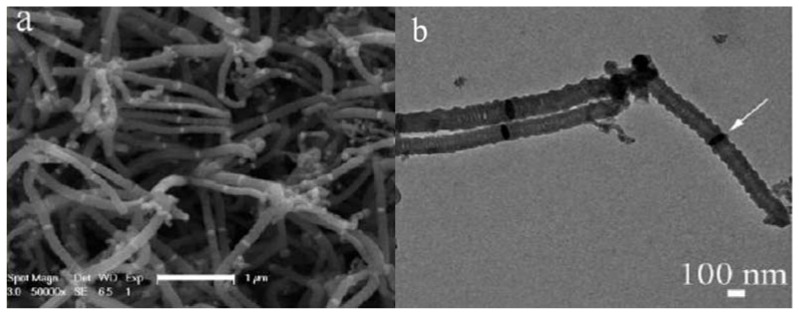
**(a)** FE-SEM and **(b)** TEM image of twin carbon nanocoils (T-CNCs) collected inside a quartz tube that was placed inside a stainless steel tube (the arrow indicates the catalyst nanoparticle) [62].

However, if the quartz tube with the Ni catalyst inside was placed in a stainless steel tube, twin carbon nanocoils (T-CNCs) (0.364 g) rather than plait-like CNCs was produced in high selectivity [62]. Figure 9 shows the FE-SEM and TEM images of the as-prepared T-CNCs. The twist and entanglement of CNCs suggest disorder and distortion of material. The T-CNCs have a novel silkworm structure, showing tight coils of very short coil pitches. Since the only difference in fabrication procedure was the inclusion of the stainless steel tube, any discrepancy in morphology of the obtained carbon species should be related to reactor modification. We found that both the obtained plait-like CNC and T-CNC materials exhibited good microwave absorbing ability (Table 1). The T-CNC materials T-CNCs are superior to plait-like CNCs in microwave absorption, showing maximum reflection loss (RL) of −36.09 dB at 17.29 GHz. The results show that reactor design undoubtedly has an impact on the selectivity and kind of carbon species produced in CCVD.

**Table 1:** Electromagnetic wave absorption properties of the plait-like CNC and T-CNC materials (Figure 8 and Figure 9).

Reactor setup	Sample	Optical RL value (dB)	$f_{m}$ (GHz) (optical RL)	Frequency range (RL<−10 and −20 dB)
quartz tube	plait-like CNCs	−20	15.96	9.01–18.0 GHz (−10 dB)
quartz tube placed inside a stainless steel tube	T-CNCs	−36.09	17.29	9.00–18.0 GHz (−10 dB) 9.00–17.2 GHz (−20 dB)

### 2.2. Effect of Catalyst Category

It is obvious that the CCVD technique is suitable for the synthesis of carbon nanomaterials in large quantity. There are two steps involved: (i) synthesis of metallic catalyst such as cobalt, iron, nickel and their alloys; (ii) pyrolysis of hydrocarbon source over catalyst at high temperatures (from 500 to 1000 °C). In the past two decades, CNTs were synthesized over Fe [63,64,65,66,67,68], Co [69,70,71,72,73,74,75], Ni [76,77,78,79,80,81] and their alloys [82,83,84,85,86,87] through the decomposition of various carbon sources. It is generally accepted that the morphology, size and yield of the obtained carbon nanomaterials are critically determined by the kind of catalyst used [88,89,90,91]. Moreover, as shown in Table 2, other inorganic materials such as Cu [92], Au [93], Cu-Cr [94], Pd-Cr-Pt [95], Cs_2_CO_3_ [96], Li_2_CO_3_ [97] and K_2_CO_3_ [97] were also used as catalysts. Across the iron-, nickel- and cobalt-based catalysts, Klinke *et al.* [98] observed that the Fe catalyst gave the highest selectivity to CNTs in the catalytic decomposition of acetylene at temperatures ranging from 580 to 1000 °C. On the other hand, Lee *et al.* [99] reported that in the synthesis of CNTs by means of acetylene pyrolysis at 950 °C, the growth rate of CNTs was dependent on the type of catalysts, and catalyst performance was in the order of Fe < Co < Ni. The average diameter of the as-synthesized CNTs followed the sequence of Fe < Co < Ni, and the crystallinity of CNTs synthesized over Fe catalyst was higher than that over Ni or Co catalyst.

**Table 2:** Products obtained in the synthesis of carbon nanomaterials by means of acetylene pyrolysis over different catalysts.

Catalyst	Product	Reference
Cu	aligned MWCNTs	[92]
Au	CNT junctions	[93]
Cu-Cr	CNTs	[94]
Pd-Cr-Pt	CNTs	[95]
Cs_2_CO_3_	CNFs and CNTs	[96]
Li_2_CO_3_	HCNFs	[97]
K_2_CO_3_	carbon nanofoam	[97]

Moreover, through the catalytic decomposition of various hydrocarbons, carbon nanomaterials other than CNTs were synthesized over iron-, nickel- and cobalt-based catalysts [100,101,102]. Over Ni nanoparticles generated through a sol-gel combustion process, Calderon-Moreno *et al.* [103] synthesized dense carbon nanospheres (CNSs) in the pyrolysis of acetylene at 700 °C. In the pyrolysis of ethylene glycol monoethyl ether (CH_3_CH_2_OCH_2_CH_2_OH) over Fe catalyst at 650–700 °C, Xi *et al.* [104] synthesized carbon nanocables and branched-nanobelts. In the co-pyrolysis of Fe(CO)_5_ and pyridine at 900–1100 °C, Bajpai *et al.* [45] produced aligned HCNT arrays in large quantity. In the Ni-catalyzed pyrolysis of acetylene containing a small amount of H_2_S, Motojima *et al.* [105] synthesized carbon microcoils (CMCs) at 700–800 °C. In terms of product yield, the nickel catalysts performed better than the iron ones for the generation of CNCs and CMCs [106,107,108,109]. So far, reports on the successful fabrication of CNCs over cobalt catalysts are rare.

In the past decade, we reported the generation of a number of nanomaterials over catalysts of Fe, Co, Ni and their alloys in high yield. We reported that HCNTs (0.413 g) (Figure 7) and HCNFs (1.582 g) (Figure 6) could be synthesized as main products in the pyrolysis of acetylene over Fe nanoparticles (0.035 g) [57,60]. The yields (as defined by $yield=\frac{m_{total}-m_{catalyst}}{m_{catalyst}}\times 100\%$) of HCNTs and HCNFs were 1,080% and 4,420%, respectively (Table 3). Moreover, due to the encapsulation of Fe nanoparticles, the HCNTs and HCNFs materials showed good magnetic properties (Table 3). Whereas, over Ni nanoparticles (rather than Fe nanoparticles), crystalline plait-like CNCs (Figure 8) or T-CNCs (Figure 9) were produced [61,62]. It is worth noting that the maximum yield of plait-like CNCs and T-CNCs was *ca.* 18,760% and 6,014%, respectively, much higher than that of HCNTs or HCNFs synthesized over Fe nanoparticles. It is clear that the growth rate of carbon nanomaterials was strongly dependent on the type of the catalysts.

**Table 3:** Yield and magnetic property of helical carbon nanofibers (HCNFs) and helical carbon nanotubes (HCNTs).

Reactor	Product yield	Product	Saturation magnetization (*M*_S_)	Coercivity (*H*_c_)
stainless steel tube	4,420%	HCNFs	4.99 emu/g	91.61 Oe
quartz tube	1,080%	HCNTs	12.11 emu/g	240.07 Oe

### 2.3. Effect of Catalyst Preparation Procedure

It is known that the dispersion level of a material has influence on its catalytic property. Unlike the fine and well dispersed particles, the large particles and aggregates were inactive for the growth of carbon nanomaterials [110]. It was reported that large surface area and well dispersion of a catalyst (*i.e.*, small in particle size) has a positive effect on the yield and size distribution of as-synthesized carbon nanomaterials [111]. In other words, in order to produce carbon nanomaterials in large quantity, one must prepare catalysts in the form of nanoparticles. For the generation of catalysts or catalyst precursors, techniques such as sol-gel approaches [60,61,62], incipient wetness impregnation [112,113,114], co-reduction of precursors [115,116], ion-exchange-precipitation [117,118,119,120], lithography [121] and ion-adsorption-precipitation [122] are often used. Since the size of catalyst nanoparticles is dependent on the preparation method, the method for catalyst preparation has an effect on the performance of the as-prepared catalyst [109,123]. In a study of Avdeeva *et al.* [124], various alkali solutions (NaOH, NH_4_OH and Na_2_CO_3_) were used to deposit Co nitrate for the formation of different Co precursors. For the generation of filamentous carbon, the best performance was found over the catalyst prepared using NaOH as precipitant [124]. In methane decomposition over Ni-Al_2_O_3_ catalysts, it was found that the catalyst prepared using NaOH as precipitant performed the best [125,126].

**Figure 10:**
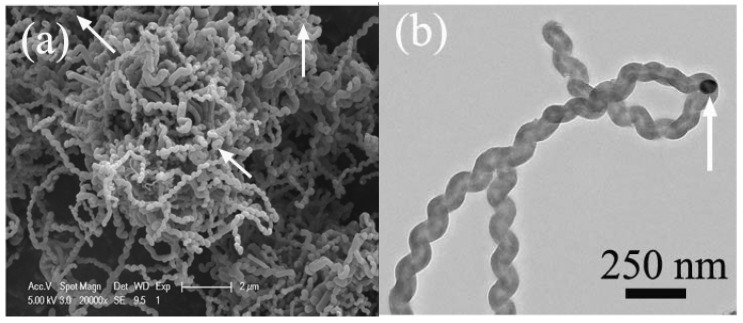
**(a)** FE-SEM and **(b)** TEM images of helical carbon nanotubes (HCNTs) synthesized over Fe nanoparticles generated by means of combined coprecipitation/hydrogen reduction method. Note: the arrows in Figure 10a indicate the opening of HCNTs and the arrow in Figure 10b indicates the catalyst nanoparticle [34].

Lately, we reported a simple, environmentally friendly and inexpensive route for the generation of HCNTs in large quantity (2.651 g). As shown in Figure 7, over Fe nanoparticles generated by a combined sol-gel/hydrogen reduction method, HCNTs (consist of two HCNTs and a catalyst nanoparticle) were synthesized as major product (selectivity = 85%; yield = 1,080%) in the pyrolysis of acetylene at 450 °C [57]. When Fe nanoparticles were fabricated using a combined coprecipitation/hydrogen reduction method instead, HCNTs were synthesized in much higher selectivity (93%) and yield (7,474%) (Figure 10a) [34], higher than any reported in the literature. The HCNTs are composed of two or three coiled nanotubes that are connected to a catalyst nanoparticle (Figure 10b). The diameters of the tubes are in the range of 50 to 100 nm, smaller than that of HCNTs shown in Figure 7 [57]. All the results confirm that the method for catalyst preparation has an effect on the shape and yield of final products.

### 2.4. Effect of Support Materials

It was found that supporting a metal or a mixture of metals on oxides, zeolites, clays, or conductive substrates would result in enhancement of catalytic activity in the synthesis of carbon nanomaterials [127,128,129,130]. It is apparent that the interaction between catalyst and support has a positive effect on the growth of carbon entities. Materials such as SiO_2_ [131,132], MgO [133,134], Al_2_O_3_ [135,136], CNTs [137,138], stainless steel [139,140], zeolite [141,142], as well as some other materials [143,144] were used as supports for the synthesis of carbon nanomaterials (Table 4). The interactions between a metal catalyst and its support can be either physical or chemical. As suggested by Vander Wal *et al.* [145], physical interaction between metal and support could have an effect on the size distribution of catalyst particles. In chemical interactions, charge transfer can take place via different pathways, e.g. through oxidation/reduction processes or acid/base (donor/acceptor) reactions. When the support is a metal oxide, oxidation/reduction reactions occur through the transfer of an oxygen atom from support to metal and *vice versa*. Acid/base interactions are linked to the Lewis acid and base characters of the materials. In the cases of metal oxides, surface anions act as Lewis base sites (electron pair donor) and cations as Lewis acid sites (electron pair acceptor). The interaction between the catalyst and its support can affect the electronic nature of the catalyst and hence have an effect on the dispersion of the metal on its support. For example, quartz, conductive glass, porous alumina and nickel plates were used by Ortega-Cervantes *et al.* [146] to support Fe and Co catalysts for the synthesis of CNTs using ethanol as carbon source. Successive growth of nanotubes was observed when conducting glass, nickel plates or porous alumina was used as support. Over the catalyst supported on nickel plate and porous alumina substrates, SWNTs were generated whereas over that supported on conducting glass, MWNTs were synthesized.

In a paper on the synthesis of double-walled CNTs over metal catalysts supported on mesoporous silica, Zhu *et al.* [147] suggested that the support was acting as template for the initial growth of CNTs. They found that when the mass fraction of Fe and Co (catalyst/support) was between 1.5% and 3%, double-walled CNTs were synthesized in high selectivity, and at mass fraction of 6% most of the products were MWNTs. Lately, we reported the controllable synthesis of CNFs, bamboo–like CNTs and chains of carbon nanospheres (CNSs) in large quantities over Fe/SnO_2_ nanoparticles generated by means of a combined sol-gel and hydrogen reduction method in the pyrolysis of acetylene at 500 °C, 600 °C and 700 °C, respectively [148]. The results demonstrated that synergistic effect of catalyst and support must be considered in CCVD processes. Nonetheless, the use of a support in the synthesis of carbon nanomaterials has its disadvantages. First, there could be the formation of undesirable intermediates. Second, further treatments are required if one has to remove the support material from the products [149]. It is likely that the separation and purification treatment would cause cost increase and damage of product. Therefore, it is good to avoid using a support or to select a support that can be easily removed without causing much damage to the product [145].

**Table 4:** Examples of supported catalysts used in CCVD synthesis.

Catalyst	Support material	Product	Reference
Ni	SiO_2_	SWNTs	[132]
Fe-Co	MgO	SWNTs	[134]
Fe	Al_2_O_3_	MWNTs	[136]
Ni	CNTs	CMCs/CNCs	[138]
Fe	stainless steel	aligned MWNTs	[141]
Co-Fe	zeolite	double-walled CNTs	[142]
Ni and Co	CaCO_3_	MWNTs	[143]
Fe	ITO	CNCs	[144]

### 2.5. Effect of Catalyst Promoters

To enhance catalytic activity and to increase selectivity to carbon nanomaterials, metals such as Cu [150], Cr [151], Mo [152], In and Sn [153] were added to catalysts as promoters. For example, Cu was added to modify the catalytic properties of Ni and Co [154,155,156]. In the cases of alloys, Cu was added as an impurity to change the crystalline state of metal ensembles [154,155] or to improve the wetting characteristics of graphite on catalyst [156]. Furthermore, it is known that Fe, Co and Ni nanoparticles provide centers for carbon nucleation and act as active sites for the decomposition of carbon species. Nonetheless, due to magnetic nature and high surface energy, nanoparticles have a high tendency to agglomerate at high temperature (generally above 700 °C). In order to retain high dispersion of Ni during C_2_H_2_ pyrolysis at 700 °C for the generation of aligned CNTs, Chen *et al.* [157] added Cr into the Ni catalyst for the formation of Ni-Cr alloy films. They found that the dispersion of Ni on the film surfaces strongly depended on the thickness ratio of Ni and Cr layers. One could optimize the density of well aligned CNTs by adjusting the thickness ratio.

As active catalysts for hydrodesulfurization, bimetallic Co-Mo materials have been studied for decades [158,159]. Recently, the catalysts were used for the selective synthesis of SWNTs [160,161,162,163]. Over Co–Mo catalysts prepared from metal acetate solutions and using alcohol as carbon source, Murakami and coworkers [164,165,166,167] generated SWNTs that were randomly or vertically aligned on quartz glass. It was reported that catalytic performance follow the order: Co(acetate)–Mo(acetate) *>* Co(nitrate)–Mo(acetate) >> Co(acetate) >> Mo(acetate). For the synthesis of SWNTs over Co-Mo using CO as carbon source, Borgna and coworkers [168,169,170,171] related the ability to produce SWNTs to the structure and composition of Co-Mo catalysts. They suggested that at optimum Co/Mo ratio, the Co species were stabilized by Co molybdate upperlayers or underlayers during reduction, and the role of Mo carbide was to provide the catalytically active metallic Co particles with active carbon. It was reported by Kitiyanan *et al.* [172] that Mo alone was not active for the synthesis of SWNTs but the copresence of Mo and Co would greatly enhance the formation of SWNTs. The results strongly suggest that the synergism of Co and Mo determines the generation of SWNTs in CCVD processes.

Furthermore, it was observed that the selectivity to HCNTs and CNCs was greatly enhanced when In and Sn were introduced to Fe catalysts. For example, Okazaki *et al.* [173] synthesized CNCs in high selectivity over Fe-In-Sn-O fine particles. By optimizing the composition ratios of Fe, In (between 10 and 33% of Fe) and Sn (less than 3.3% of Fe), CNCs could be grown in high selectivity. Moreover, using Fe and indium tin oxide (ITO) as catalysts, Pan *et al.* [174] selectively produced carbon tubule nanocoils in CCVD of acetylene. It was reported that the selectivity to CNCs was determined by the molar ratio of Sn and In, and the growth of CNTs was related to Fe whereas the helical growth to ITO.

**Figure 11:**
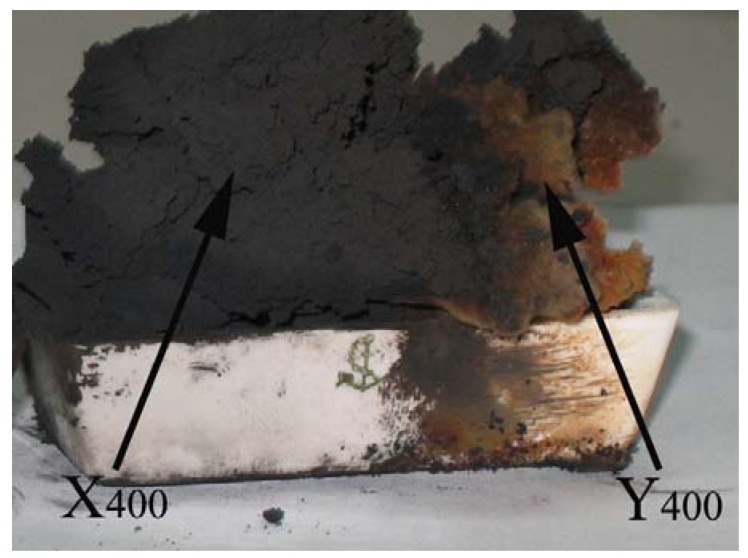
Photo of X_400_ and Y_400_ deposited on a ceramic plate [175].

**Figure 12:**
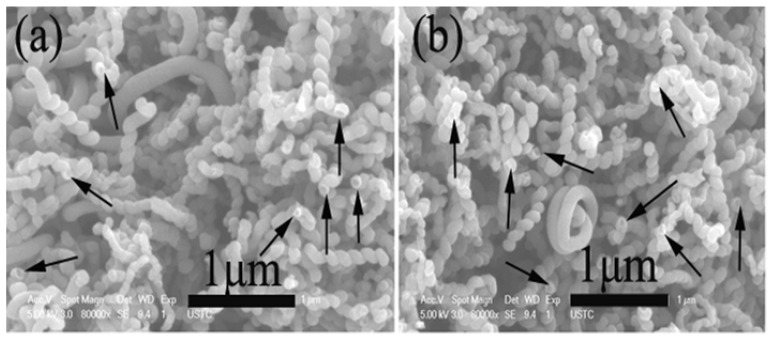
(**a-b**) FE-SEM images of X_400_ at different magnifications (the arrows indicate the opening of HCNTs) [175].

Recently, we reported the effect of introducing Cu to Fe_2_O_3_ precursor on the pyrolysis of acetylene [175]. Over Fe-Cu nanoparticles derived from sol-gel synthesis followed by hydrogen reduction at 400 °C, HCNTs and CNBs were produced in large quantities at 450 °C. As indicated in Figure 11, the black product was labeled X_400_ while the brown one Y_400_. The FE-SEM images of X_400_ show that the selectivity to HCNTs (*ca.* 85%) is high (Figure 12). Unlike the case of generating double-HCNTs over Fe at 450 °C (Figure 7) [62], plait-like HCNTs are produced. One can see openings of CNTs as indicated by arrows in Figure 12.

**Figure 13:**
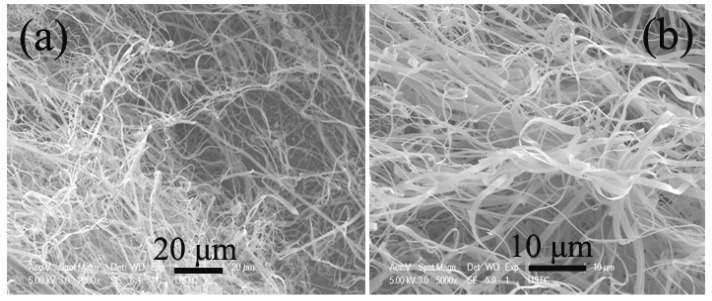
(a-b) FE-SEM images of Y_400_ at different magnifications [175].

The FE-SEM images of Y_400_ are shown in Figure 13. The majority of the product is CNBs (*ca.* 90%). The amount of Y_400_ collected was 3.468 g, slightly higher than one-third of the total mass (9.504 g of X_400_ and Y_400_). In other words, the yield of CNBs was *ca.* 9,867%. The results of a series of comparison experiments designed to investigate the influence of synthesis conditions confirmed that the presence of Cu in the catalyst is crucial for CNBs fabrication.

### 2.6. Effect of Reduction Temperature of Catalyst Precursors and Presence of Hydrogen in Carbon Source

As mentioned above, the catalyst precursors were synthesized and reduced in hydrogen at different temperatures to generate the corresponding metal catalysts (Fe, Co or Ni) [57,62,175]. It is noted that the temperature adopted for the reduction of precursors has an effect on the final products [176,177,178,179]. Reduction is an important process, which determines the energetic state and the size of the metal particles. Piao *et al.* [180] studied the effect of reduction temperature of catalyst precursor (580, 700 and 800 °C) on the synthesis of CNTs over Ni/Al_2_O_3_ at 575 °C using a feed of CH_4_:N_2_ = 1:2. They found that the amount, morphology and the growth rate of CNTs depended on the reduction temperature. In the CVD of methane over Co-Mo/Al_2_O_3_ catalysts in a stainless steel fixed-bed reactor at 700 °C, Chai *et al.* [181] observed that the reduction temperature had great impact on CNT selectivity and diameter uniformity. Their results showed that an increase in reduction temperature could produce metal particles that are slightly larger in average diameter and wider in diameter distribution. Also, with an increase of reduction temperature of catalyst precursor, better graphitization of CNTs was observed.

The effect of H_2_ presence during pyrolysis process on as-synthesized carbon nanomaterials can be significant. Signore *et al.* [178] found that CNTs could be synthesized at 700 °C over Fe catalyst in C_2_H_2_/H_2_ flows (ranging from 5/95 to 30/70), and when C_2_H_2_/H_2_ flow rate was greater than 20% vertically aligned CNTs were fabricated in 7 min of deposition time. Also, in the synthesis of carbon coils in H_2_/C_2_H_2_ flows over Ni catalysts, Chen *et al.* [182] observed that at low H_2_ flow rate (*i.e.*, low H_2_/C_2_H_2_ flow ratio), there was the formation of irregular carbon coils (different in coil diameters and irregular in coil diameters) as well as the formation of single coils, straight fibers and irregular fibers. At high H_2_ flow rate (*i.e.*, high H_2_/C_2_H_2_ flow ratio), there was the preferable formation of short coils, and the yield of regular coils was poor. It is apparent that the amount of hydrogen in acetylene affects the kind of hydrocarbon intermediates being formed, and thus affects the growth characteristic of carbon coils. Moreover, under the conditions of: catalyst = Ni, reaction temperature = 770 °C, reaction time = 60min, and gas flow: C_2_H_2_ = 83 sccm, N_2_ = 400 sccm, H_2_S/H_2_ = 70 sccm, Chen *et al.* [183] observed the formation of CMCs. The authors reported that when H_2_ flow rate was below 75 sccm, only carbon powder was produced. Between H_2_ flow rate of 100 and 250 sccm, there was steep increase of circular CMCs. Maximum selectivity (85%) to CMCs was attained at 250 sccm, and selectivity to CMCs declined to 60% at H_2_ flow rate of 430 sccm.

**Figure 14:**
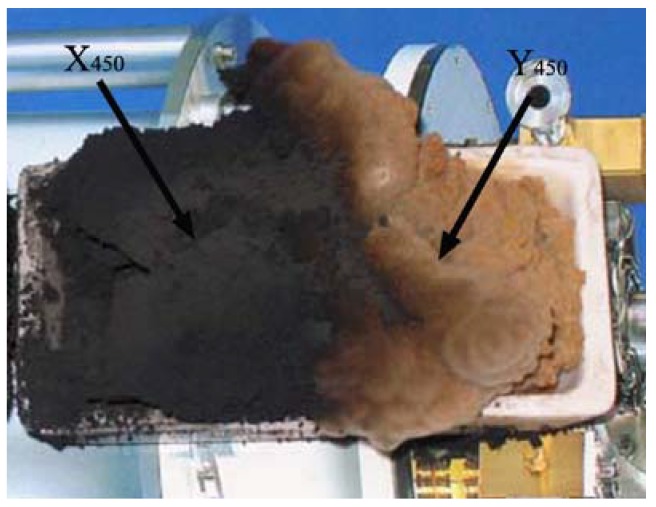
Photo of X_450_ and Y_450_ [175].

As mentioned above, over the Fe-Cu nanoparticles (0.037 g) derived from sol-gel synthesis of Fe_2_O_3_/CuO followed by hydrogen reduction at 400 °C, HCNTs and CNBs (9.504 g) were synthesized simultaneously in large quantities in acetylene decomposition at 450 °C [175]. The yield of carbon species is extremely high, up to *ca.* 27,307% (Figure 11). The amount of Y_400_ (CNBs) collected was 3.468 g (corresponding to a yield of *ca.* 9,867%) slightly higher than one-third of the total mass. When reduction temperature of Fe_2_O_3_/CuO was 450 °C (other experimental conditions were kept equal), the amount of black and brown products (denoted as X_450_ and Y_450_ as indicated in Figure 14) collected was 3.860 g (corresponding to a yield of around 10,994%), much lower than that of X_400_ and Y_400_. Compared to Y_400_, Y_450_ was deeper in color and higher in amount. After separation, 1.831 g of Y_450_ was collected (almost half of the total mass), giving a CNB yield of *ca.* 5,163%, much lower than that of Y_400_.

**Figure 15:**
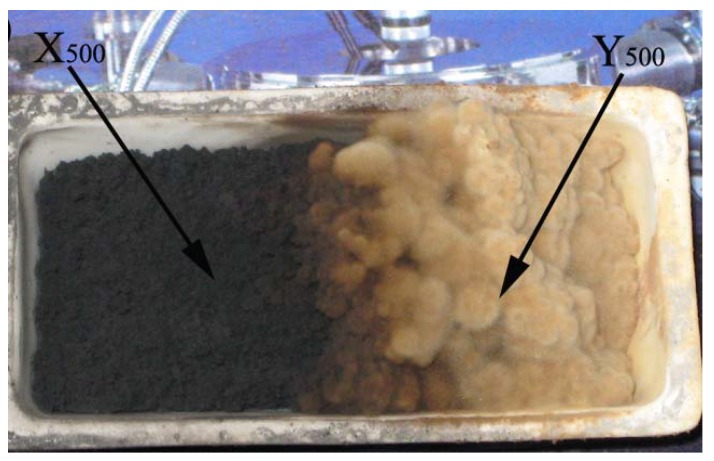
Photo of X_500_ and Y_500_ [175].

FE-SEM and TEM observations of X_450_ indicated that HCNTs and worm-like CNTs were produced in large amounts. The selectivity to HCNTs in the case of X_450_ is poorer than that in the case of X_400_. The FE-SEM and TEM investigations of Y_450_ showed that the selectivity to CNBs was up to *ca.* 90%. The CNBs are wider than those observed in Y_400_. It is hence deduced that the reduction temperature of catalyst precursor has profound effects on the yield and morphology of products. In order to confirm such a view, the Fe_2_O_3_/CuO powder was reduced at 500 °C. After the completion of the pyrolysis process, 2.342 g of black and brown products (denoted as X_500_ and Y_500_) was collected (Figure 15), giving a yield of *ca.* 6,631%. In contrast to X_400_ (Figure 11) and X_450_ (Figure 14), there were CNTs of low helicity (L-HCNTs) in X_500_ and the content of CNBs in Y_500_ was at least up to *ca.* 90%. Compared to that of Y_400_ and Y_450_, the content of Y_500_ (2.342 g) was high. After separation, 1.263 g (exceeding half of the total mass) of Y_500_ was obtained, and the yield of CNBs was up to 3,530%. It is clear that a rise of reduction temperature from 400 to 500 °C favors the generation of CNBs but lowers the total yield of carbon nanomaterials. Since the decomposition of acetylene was conducted at equal temperature (450 °C) in the three cases, any discrepancy in yield and morphology of carbon species should be related to the reduction temperature of catalyst precursor.

It was also observed that the temperature for NiO reduction has a profound influence on the morphology and yield of as-synthesized plait-like CNCs [60]. If NiO was reduced in H_2_ at 375 °C for 1 h, 3.705 g (corresponding to a yield of *ca.*18,760%) of crystalline plait-like CNCs was synthesized in the pyrolysis of acetylene at 425 °C. The plait-like CNCs showed good ability for microwave absorption. If the temperature for NiO reduction was 400 °C (rather than 375 °C) and with the other experimental conditions kept equal, the decomposition of acetylene became very fast (completion in 12 min), and the observed carbon yield was *ca.* 4.198 g (corresponding to a carbon yield of *ca.* 21,270%). The TEM and FE-SEM investigations showed that there was a dense population of single helicals among the product and the amount of plait-like CNCs was low. In other words, with a rise of temperature (from 375 to 400 °C) for the reduction of NiO precursor, the selectivity to single CNCs became high and that to plait-like CNCs low.

We also investigated the effect of hydrogen flow rate during the pyrolysis process on morphology, yield, and magnetic property of carbon products. As pointed out before [34], over Fe nanoparticles derived from coprecipitation/hydrogen reduction method, HCNTs (2.651 g) (as shown in Figure 12) could be synthesized in large-scale in the pyrolysis of acetylene at 450 °C and the yield of HCNTs is *ca.* 7,474%. TEM and FE-SEM investigations indicated that the as-synthesized HCNTs showed high helicity (H-HCNTs) in comparison to those of HCNTs reported by Bajpai [44], Hou *et al.* [46], and Tang *et al*. [57]. Moreover, the HCNT material showed good microwave absorbing ability [184]. If hydrogen was introduced to the reaction tube at low rate (C_2_H_2_/H_2_ = 5:1) and the other experimental conditions were kept unchanged, 0.526 g of L-HCNTs (low helicity of HCNTs) was collected. The corresponding yield was *ca.* 1,403%, much lower than that of H-HCNTs (Figure 12). A comparison between the H-HCNT and L-HCNT material showed that the latter exhibited better microwave absorption properties than the former [184]. In general, by controlled introduction of hydrogen into the reaction tube, H-HCNTs, L-HCNTs and worm-like CNTs could be fabricated in the catalytic decomposition of acetylene over Fe nanoparticles generated by a combined coprecipitation/hydrogen reduction method. When the H_2_ flow rate was enhanced (C_2_H_2_/H_2_ = 5:3), 0.312 g of worm-like CNTs (selectivity = 88%) was obtained, and the corresponding yield of carbon species is *ca.* 891%, much lower than that of H-HCNTs and L-HCNTs. Compared to that of H-HCNTs and L-HCNTs, the helicity of worm-like CNTs can be considered as zero. Through FE-SEM and TEM observations of the obtained samples, we found that the size of catalyst nanoparticles (*ca.* 200 nm) of worm-like CNTs is much larger than that of H-HCNTs and L-HCNTs (*ca.* 40 and 60 nm, respectively). It is clear that the introduction of hydrogen and rate of hydrogen introduction have an effect on CNT helicity as well as on the size of entrapped catalyst nanoparticles. In terms of yield, morphology and helicity of CNTs products, there are distinct variations among the obtained H-HCNTs, L-HCNTs and worm-like CNTs. Moreover, as shown in Table 5, the as-synthesized worm-like CNT material is superior to the H-HCNT and L-HCNT material in microwave absorption.

**Table 5:** Electromagnetic wave absorption properties of as-synthesized H-HCNT, L-HCNT and worm-like CNT samples.

Sample	Optical RL value (dB)	$d_{m}$ (mm) (RL<−20 dB)	$f_{m}$ (GHz) (optical RL)	Frequency range (GHz) (RL<−20 dB)
H-HCNTs	−8.25	–	11.8	–
L-HCNTs	−25.78	2.0–3.0	7.18	7.18–10.68
worm-like CNTs	−26.39	2.0–3.0	7.71	7.5–10.7

### 2.7. Effect of Reaction Temperature

In previous studies, it was found that the yield and structure of as-synthesized carbon materials were dependent on the reaction temperature [185,186,187,188,189]. For example: Li *et al.* [190] reported the synthesis of MWNTs over Ni/Al catalyst in methane decomposition at 450–650 °C. They found that the yield of MWNTs increased with rise of pyrolysis temperature and this effect became more apparent at high temperatures. They reported that CNTs poor in structure and low in crystallinity were formed at low temperatures due to the low catalytic activity of nickel and poor reactivity of carbon atoms. The effect of reaction temperature was also reported by Singh *et al.* [191]. They found that at 550 °C, the yield of MWNTs over an iron catalyst was low. When the reaction temperature was raised to 590, 740 or 850 °C, film with aligned CNTs was formed. According to the results of TEM investigation, the diameters of the nanotubes increased with rise of reaction temperature. At 940 °C, the quantity of CNTs decreased; alignment was lost and a large number of encapsulated particles were formed. For the generation of HCNFs, Tang *et al.* [60] achieved high selectivity to coiled nanofibers in acetylene pyrolysis over Fe nanoparticles at 450 °C in a stainless steel tube. At 700 °C, fibers with straight and/or irregular shapes became major product. The amount of carbon products generated at 700 °C was 15.753 g, much higher than that of HCNFs generated at 450 °C. The results again indicated that the yield and morphology of CNFs was dependent on reaction temperature.

In CMC synthesis, Chen *et al.* [182] found that coil diameters and morphology of carbon coils and diameter of carbon fibers that built up the carbon coils were significantly affected by reaction temperature. The regular carbon coils with an average coil diameter of about 5 μm and an average fiber diameter of about 0.7 μm were obtained at temperatures between 760–790 °C. Below 760 °C, the formation of CMCs was insignificant. At 700 °C, only noodle-like twin fibers were formed whereas at 740 °C, a smaller number of carbon coils was formed, showing coil diameters and coil pitches larger than those of the CMCs obtained in the 760–790 °C range. At higher temperature such as 870 °C, a small amount of irregular carbon coils with larger coil diameters were produced. Similar phenomena were observed in the synthesis of T-CNCs [62] and plait-like CNCs [61]. When the reaction temperature was changed from 415 to 425 °C (other conditions unchanged), T-CNCs (2.41 g) larger in length and diameter (*ca.* 160 nm) were obtained over Ni nanoparticles. We observed that the coils were composed of small nanocoils of very small diameter. As compared to that of 0.364 g at 415 °C, there is a significant increase in T-CNC yield at 425 °C. On the other hand, 3.302 g of plait-like CNCs were synthesized over Ni nanoparticles in acetylene pyrolysis at 415 °C. If the decomposition temperature was changed from 415 to 425 °C, CNCs production was *ca.* 3.705 g, corresponding to a yield of *ca.* 18,759.8%, much higher than that of plait-like CNCs synthesized at 415 °C. Similar to the case of synthesis at 415 °C (Figure 8a), plait-like CNCs of high purity were obtained at 425 °C, indicating that a change of pyrolysis temperature from 415 to 425 °C only increases the yield of plait-like CNCs but has little effect on the morphology of the CNCs sample. Moreover, we found that the pyrolysis temperature had a significant effect on microwave absorbing ability of carbon products. Compared to plait-like CNCs synthesized at 415 °C, the plait-like CNCs synthesized at 425 °C showed a slight decrease in microwave absorbing ability.

**Figure 16:**
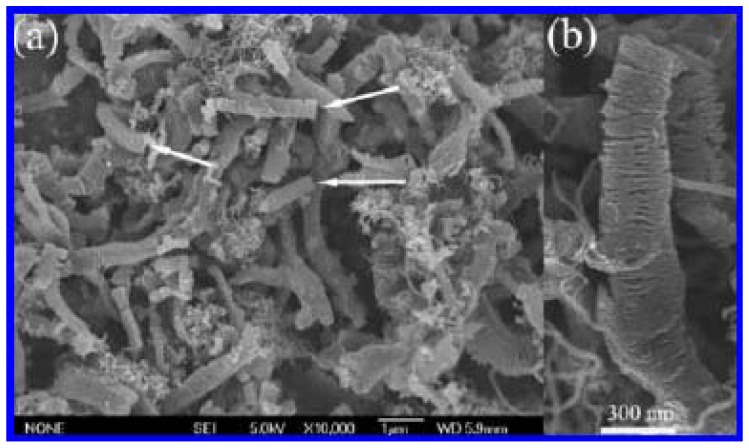
**(a)** Low and **(b)** high magnification of FE-SEM images of samples synthesized at 350 °C. The arrows indicate the “clear-cut” ends of CNRs [192].

In the synthesis of CNRs through the copyrolysis of C_6_H_6_ and C_5_H_6_ over cocatalysis of Fe and Mg, Zou *et al.* [101] observed no activity below 400 °C. At 500 °C, short CNRs were produced. In the 600–700 °C range, longer CNRs were obtained in large quantity (*ca.* 90% yield). At 800 °C, carbon particles were formed. Over Ni nanoparticles derived from sol-gel synthesis followed by hydrogen reduction, we synthesized CNRs (0.386 g) composed of carbon nanoflakes through the catalytic decomposition of benzene at temperature as low as 350 °C [192]. The yield of CNRs was *ca.* 1,537%. There were nanorods of several microns in length and 200–400 nm in diameter (Figure 16). In Figure 16b, one can observe that the CNRs are composed of nanoflakes. It is known that interaction between graphitic layers is weak, and rupture of this kind of CNRs should be easy. We found “clear-cut” ends of CNRs different from those of CNTs, CNCs, and CNFs, and attributed the phenomenon to the fracture of flake-composed CNRs. Based on the FE-SEM images, we estimated a CNR selectivity of *ca.* 90%; the rest are coiled nanowires and nanotubes of irregular morphology. When the decomposition temperature was 460 °C, *ca.* 1.103 g of CNRs was collected, corresponding to *ca.* 4,579% yield of CNRs, much higher than that of CNRs obtained at 350 °C. With a change of decomposition temperature from 350 to 460 °C, there was an increase of CNR yield and a change in CNR morphology: the nanorods of CNRs obtained at 460 °C became curly in shape and bigger in diameter (*ca.* 500 nm). A closer TEM investigation revealed that the curly nanorods were also composed of carbon nanoflakes. However, at 550 °C, the majority of carbon species were MWNTs with inner diameter *ca.* 5 nm and outer diameter ranging from 15 to 40 nm; the selectivity to CNTs was high, up to *ca.* 88%. All the results demonstrated that the reaction temperature is an important factor in CNR formation.

### 2.8. Extra Effects

Many studies revealed that carbon source [180,193,194] and added gas such as H_2_S and thiophene [195,196,197] are factors that influence the yield and quality of final products. The most widely used carbon sources are acetylene, methane, ethylene, ethanol, benzene and toluene. Due to the thermodynamic properties and chemical structures of the organic molecules, the hydrocarbon intermediates generated on catalyst surface have a significant effect on the morphology of final products. Using methane, hexane, cyclohexane, benzene, naphthalene and anthracene as carbon source in the synthesis of CNTs over MgO-supported Fe catalyst (500–850 °C), Li *et al.* [198] came to the conclusion that chemical structures rather than thermodynamic properties of hydrocarbons determine the morphology of products. Also, it was found that aromatic molecules are better for the growth of SWNTs, whereas the use of aliphatic molecules usually result in the formation of MWNTs or species with non-tubular structures. Using chlorinated benzene, C_6_H_6-x_Cl_x_ (x = 0–3), as carbon precursors, Lv *et al.* [199] produced permalloy (FeNi) nanowires micrometer in length inside thin-walled CNTs. They found that FeNi content and hollow degrees of CNTs increased in the order of C_6_H_6_ (x = 0) < C_6_H_5_Cl (x = 1) < C_6_H_4_Cl_2_ DCB (x = 2) < C_6_H_3_Cl_3_ (x = 3). The authors attributed the formation of thin-walled CNTs to “carbon-rich and hydrogen-deficient” condition generated as a result of Cl combination with reactive hydrogen. Under such condition, the formation of sp^2^-like graphitic structure that is critical for the formation of thin-walled CNTs became favorable.

Moreover, gas such as H_2_S and thiophene is often added to the carbon source during pyrolysis to optimize the quality of carbon nanomaterials. Generally, CNTs prepared through the pyrolysis of hydrocarbon source at high temperatures (500 to 1000 °C) have straight or randomly curled morphology. In most cases, CNCs were reported as a by-product. In the past few years, efforts have been put in to synthesize regular CNCs in high selectivity. Motojima and coworkers [200,201] detected regular coiled carbon fibers of micron size in CCVD processes. In the experiments, carbon atoms coming from the pyrolysis of acetylene or propane deposited on micron-sized Ni particles in the presence of thiophene. Recently, using commercial acetylene as carbon source, nickel plate as catalyst and phosphorous compound as additive, Zhao and Shen [202] synthesized CNCs with high selectivity. Adding a small amount of H_2_S into acetylene, Chen and coworkers [203] synthesized twisting CNCs in high selectivity over Ni/Al_2_O_3_ at 760 °C. Again by the addition of a small amount of H_2_S in acetylene, the authors synthesized CMCs over Ni in the 700–800 °C range [204]. Overall, the selectivity to carbon coils could be greatly enhanced by adding sulfur or phosphorus into the carbon source.

## 3. Conclusions

Since the landmark paper of Ijima published in 1991, carbon nanotubes are designated as one of the most attractive materials for a wide range of potential applications. In the past two decades, there have been advances in the production of carbon nanotubes in terms of quality as well as quantity. In the course of it, carbon nanomaterials of other structures, such as carbon nanocoils, carbon nanorods, carbon nanobelts and grapheme, were detected and investigated. Contributions have been made toward the mass synthesis and understanding of magnetic and microwave absorption properties of carbon nanomaterials. It is observed that by means of CVD over catalysts made of transition metals, it is possible to selectively generate carbon nanomaterials in high quantity. Among the catalysts, those based on iron, cobalt, nickel and their alloys perform well. As carbon sources, acetylene, methane, ethanol, ethylene, benzene and toluene are widely used. It is known that factors such as reactor design, preparation method of catalysts, catalyst category, support, catalyst additive, precursor reduction temperature, pyrolysis temperature, carbon source, additive to carbon source, and reaction time have impact on the growth of carbon nanomaterials. By means of careful manipulation of these factors, one can selectively produce a desired carbon nanomaterial in large scale. For wide utilization of carbon nanomaterials, research on this aspect remains challenging.
